# Draft Genome Sequence and Annotation of Acinetobacter soli AS15, Isolated from an Irish Hospital

**DOI:** 10.1128/MRA.00611-21

**Published:** 2021-10-07

**Authors:** Lawa Shaban, Anna S. Ershova, Fergal J. Hamrock, Ali Shaibah, Maha M. Sulimani, Mohammad R. Amin, Jennifer N. Russell, Rushiil Ravichandran, Kirsten Schaffer, Marta Martins, Andrew D. S. Cameron, Carsten Kröger

**Affiliations:** a Department of Microbiology, School of Genetics and Microbiology, Moyne Institute of Preventive Medicine, Trinity College Dublin, Dublin, Ireland; b Department of Biology, University of Regina, Regina, Saskatchewan, Canada; c Institute for Microbial Systems and Society, University of Regina, Regina, Saskatchewan, Canada; d Department of Microbiology, Noakhali Science and Technology University, Noakhali, Bangladesh; e Department of Microbiology, St. Vincent’s University Hospital, School of Medicine, University College Dublin, Dublin, Ireland; University of Arizona

## Abstract

We report the draft genome sequence of Acinetobacter soli AS15, which was isolated in 2018 from a rectal screen of a patient at St. Vincent’s University Hospital (Dublin, Ireland). The draft genome sequence is 3,589,002 bp and was assembled into 82 contigs.

## ANNOUNCEMENT

In 2008, a novel Acinetobacter species, Acinetobacter soli, was discovered in forest soil in the Republic of Korea (1). A few studies have since reported that *A*. *soli* was the cause of outbreaks in hospitals. The species was isolated from bloodstream infections in Brazil in a neonatal intensive care unit and in a tertiary hospital in Japan (2, 3) and has been reported to carry carbapenemase genes, including *bla*_oxa-58_, *bla*_NDM-1_, and *bla*_TMB-2_ (4–6). We report the draft genome sequence of *A. soli* AS15, which was isolated from a rectal screen of a patient in St. Vincent’s University Hospital (Dublin, Ireland). Ethics approval for the investigation of bacterial isolates from clinical samples was obtained from St. Vincent’s University Hospital. There are only 36 *A*. *soli* chromosome assemblies in GenBank, of which 2 are completed (accessed 9 July 2021). One strain isolated in Europe has been sequenced; thus, our *A*. *soli* AS15 draft genome sequence represents a rare isolate from Europe. The specimen was cultured on a blood agar plate at 37°C and identified as an Acinetobacter species by matrix-assisted laser desorption ionization–time of flight mass spectrometry (MALDI-TOF/MS) at St. Vincent’s Hospital. The colony appearance of *A*. *soli* AS15 grown on Lennox broth agar plates is round, smooth, opaque, convex, and 1 to 3 mm in diameter after incubation at 37°C for 16 h. For draft genome sequencing, genomic DNA of *A. soli* AS15 was isolated using the DNeasy UltraClean microbial kit (Qiagen) from a 5-ml (L broth) overnight culture inoculated with a single colony grown on L agar. Preparation of the DNA library was carried out using the Illumina DNA prep kit (20018704) and sequencing using Illumina MiSeq technology (2 × 150-bp paired-end reads; MiSeq reagent kit v2 [300 cycles], MS-102-2002), which produced 931,684 reads. The samples were quality inspected using FastQC v0.11.8 and MultiQC v1.7 (7) and were trimmed and quality filtered (Q30) using Trimmomatic v0.39 (8), with a minimum length of 100 bp, trimming of the first 5 bp and the last 30 bp (if below the quality threshold), and trimming of reads whose average quality dropped below 30 over a 10-bp window. The draft sequence *de novo* assembly was performed using SPAdes v3.15.1 (--careful function) (9), and the assembly quality was assessed using QUAST v5.1.0rc1 (10). Species identification was carried out using JSpeciesWS v3.8.2 (default parameters) (11). The draft genome sequence is comprised of 3,589,002 bp and was assembled into 82 contigs (contig size, >500 bp; *N*_50_, 93,566 bp; GC content, 42.75%; genome coverage, 41×). The average nucleotide identity, calculated using the BLAST algorithm (ANIb) and the MUMmer alignment tool (ANIm), returned >97.6% and >98.58% sequence identity, respectively, with the reference strain, *A. soli* GFJ2 (>87% aligned nucleotides). Because the average nucleotide identity was >95% with the reference, we assigned the new strain as a member of the species *A*. *soli*. Genome annotation was carried out during the sequence submission to NCBI using the Prokaryotic Genome Annotation Pipeline v5.2 (default parameters) (12), which predicted the presence of 3,521 genes, including 3,464 coding sequences, 46 tRNA genes, 74 pseudogenes, and three CRISPR arrays.

### Data availability.

The raw reads used for the draft genome sequence assembly were deposited in the Sequence Read Archive (SRA) under accession number SRR14887741 and BioProject number PRJNA740158. This whole-genome shotgun project has been deposited at DDBJ/ENA/GenBank under the accession number JAHRBJ000000000. The version described in this paper is version JAHRBJ010000000.
